# Small size fullerenol nanoparticles suppress lung metastasis of breast cancer cell by disrupting actin dynamics

**DOI:** 10.1186/s12951-018-0380-z

**Published:** 2018-06-23

**Authors:** Yanxia Qin, Kui Chen, Weihong Gu, Xinghua Dong, Ruihong Lei, Yanan Chang, Xue Bai, Shibo Xia, Li Zeng, Jiaxin Zhang, Sihan Ma, Juan Li, Shan Li, Gengmei Xing

**Affiliations:** 1 School of Biology and Biological Engineering, South China University of Technology, Guangzhou, 510006 China; 2 CAS Key Laboratory for Biomedical Effects of Nanomaterial & Nanosafety, Institute of High Energy Physics, Chinese Academy of Sciences, Beijing, 100049 China; 3 University of Chinese Academy of Sciences, Beijing, 100049 People’s Republic of China

**Keywords:** Fullerenol nanoparticles, Metastasis, Actin dynamics, Young’s modulus, Filopodia

## Abstract

**Background:**

Tumor metastasis is the primary cause of mortality in cancer patients. Migratory breast cancer cells in lymphatic and blood vessels seek new sites and form metastatic colonies in the lung and bone, and then these cancer cells often wreak considerable havoc. With advances in nanotechnology, nanomaterials and nanotechnologies are widely applied in tumor therapy. In this paper, small size fullerenol nanoparticles, which are separated by isoelectric focusing electrophoresis (IFE) for discrepancy of isoelectric point (*p*I), are used in the study of tumor metastasis.

**Results:**

In this study, the commendable inhibition of tumor metastasis was uncovered by intravenous injection of purified fullerenol fraction with special surface charge and functional groups, which was separated by IFE for discrepancy of *p*I. By investigating the actin dynamics in several cancer cell lines, we found these small size fullerenol nanoparticles disturbed actin dynamics. Young’s modulus detection and cell migration assays revealed that fullerenol lowered stiffness and restrained migration of breast cancer cells. Filopodia, the main supporting structures of actin bundles, are important for cell motility and adhesion. Scanning electron microscopy showed that fullerenol reduced the number and length of filopodia. Simultaneously, the inhibition of integrin to form clusters on filopodias, which was likely induced by reorganizing of actin cytoskeleton, impacted cancer cell adhesion and motility.

**Conclusions:**

With intravenous injection of these fullerenol nanoparticles, tumor metastasis is well inhibited in vivo. The underlying mechanism most likely to be attributed to the effect of fullerenol nanoparticles on disturbing actin dynamics. With the disordered actin fiber, cell function is varied, including decreased cell stiffness, reduced filopodia formation, and inactivated integrin.

**Electronic supplementary material:**

The online version of this article (10.1186/s12951-018-0380-z) contains supplementary material, which is available to authorized users.

## Background

Metastasis is responsible for over 80% of cancer mortality [1]. With recent advances in nanotechnology, researchers have begun to utilize nanomaterials and nanotechnologies to prevent malignant cells from leaving the primary tumor mass and traveling through the blood and lymphatic vessels to found new colonies, finally leading to metastasis [2–5]. Targeting the molecular and cellular events of tumor metastases could be a useful oncotherapy to prevent cancer cell dissemination.

Some researchers have found that actin cytoskeletal reorganization is highly correlated with tumor progression [6–8]. The overall mechanism of tumor metastasis is a transition in which cancer cells go from immotile to motile with dynamic actin assembly and aggregation. The correlative regulated proteins of the actin cytoskeleton could therefore be targeted to prevent metastasis [9]. Many studies have revealed that carbon nanomaterials can affect actin cytoskeleton remodeling, ultimately affecting cell migration. Shams et al. reported that single-wall carbon nanotubes (SWCNTs) directly affect actin cytoskeleton organization by interacting with G-actin monomers [10]. Tian et al. demonstrated that graphene oxide (GO) nanosheets disrupt the actin cytoskeleton via insertion into the interstrand gap of actin tetramers and can slow cell migration [11]. Metallofullerenol inhibits the development of tumor tissue [12, 13], while fullerenol, a structural analogue of metallofullerenol, exhibited a weaker ability to control tumor development. In other hand, the pure carbon-fullerenol has excellent water solubility and superior biocompatibility [14]. The addition of surface chemical structures including hemiketal (RO–C–OH), ketone (C=O), cycloxygen (C–O–C) and hydroxyl (C–OH) groups allow further tuning of fullerenol’s biological effects [15–18]. Furthermore, we previously reported that fullerenol fraction with *p*I = 2.81 have a surface electric charge of − 1.913 ± 0.008 q (× 10^−6^ C), and the percentages of C–OH and C=O on the carbon cages were 16.14 ± 0.60 and 17.55 ± 0.69, respectively [19], C–OH and C=O acted as acceptors or donors that facilitate the binding of the donator acceptor group (–COOH, –NH, or –OH) of actin to form hydrogen bonds, which allow them to affect actin cytoskeleton and thus restrain cell migration.

Here, we purified the fullerenol fraction (*p*I = 2.81) to further evaluate the inhibition effect on tumor metastasis. The AFM result showed that these fullerenol nanoparticles performed small size within 2 nm and behaved no significant effect on cell viability and apoptosis. Intravenous administration of these small size fullerenol, the lung metastasis was well inhibited in mouse model. Cancer cell migration and invasive capacities were also suppressed after fullerenol treatment. We performed atomic force microscopy (AFM) to evaluate Young’s modulus values of cancer cells and observed decreased stiffness following fullerenol exposure. The variability of cellular stiffness was accompanied by altered equilibria of F-actin and G-actin and actin cytoskeleton reorganization in cancer cells. Finally, we observed fewer filopodia and redistributed integrin in the cytoplasm of fullerenol-treated cancer cells. Based on these results, we suggest that fullerenol disrupts actin dynamic assembly to arrest cancer cell migration and invasion, thus reducing the ability of breast cancer cells to travel through the vasculature and form new colonies in the lung.

## Methods

### Materials

The cancer cell lines (MCF-7 and MDA-MB-231 cells) were obtained from China Infrastructure of Cell Line Resource (Beijing, China). The MDA-MB-231 cell line with stable luciferase expression (MDA-MB-231-luc cells) was purchased from Zhong Qiao Xin Zhou Biotechnology Co, Ltd (Shanghai, China). The MCF-10A cells were supported by Institute of Biophysics, Chinese Academy of science (Beijing, China). MCF-7 cells were cultured in DMEM high glucose medium supplemented with 10% fetal bovine serum (FBS, PAN, Germany), and MDA-MB-231 cells were cultured in L15 medium containing 10% FBS at 37 °C without CO_2_. The MCF-10A cells were cultured in DMEM/F12 medium containing 5% horse serum, 10 μg/mL insulin, 0.5 μg/mL hydrocortisone, 20 ng/mL hEGF and 100 ng/mL cholera toxin.

BALB/c nude mice (5 weeks, 14–16 g) were purchased from Beijing Vital River Laboratory Animal Technology Co., Ltd (Beijing, China). Mice were fed for 5 days in order to adapt to the environment prior to the experiment. All the animal experiments were performed with the approval of the Institutional Animal Care and Use Committee at the Institute of Tumors at the Chinese Academy of Medical Sciences.

## Experimental methods

### Characterization of fullerenol

The special fullerenol fraction was purified by IFE as described in our previous work [14]. Fullerenol nanoparticles were dissolved in phosphate buffered saline (PBS, pH 7.4) and sonicated for 10 min to fully dissolved and dispersed. Particle size was detected by atomic force microscopy (Agilent 5500, USA), and zeta potentials of fullerenol nanoparticles were evaluated by dynamic light scattering spectrophotometer (Brookhaven NanoBrook Omni, USA) six times for each case.

### Cytotoxicity assay

The human breast cancer cells (MCF-7, and MDA-MB-231) were suspended and 5 × 10^3^ cells were seeded in each well of 96-well plate. After 24 h, fullerenol at dose of 12.5, 25, 50, 100, 200 μg/mL were added to treat the cells for 24, 48, and 72 h. Commercial Cell Counting-8 (CCK-8, Dojindo, Japan) was used to assess the cell viability. Moreover, the live/dead cells were visualized by Calcein-AM/PI staining. The images were acquired using fluorescence inverted microscope (Olympus IX71, Japan).

### Detection of apoptosis

Apoptosis was measured with Annexin V-FITC/PI apoptosis detection kits (Invitrogen, USA). Cells were treated with fullerenol at various concentration for 24 h. Then, cells were washed with PBS and suspended in 100 μL staining buffer containing 5 μL Annexin V-FITC and 5 μL PI for 30 min. The percentage of apoptotic cells was measured by flow cytometer (FACS, Accuri C6 BD Biosciences, USA). To detect mitochondrial membrane potential and further verify the early apoptosis, cells were treated by Mitochondrial membrane potential assay kit (Beyotime, China) according to the manufacturer’s instructions, JC-1 was used to analyze the percentage of early apoptosis, and imaging was performed on Laser Scanning Confocal Microscope (Nikon, Tokyo, Japan).

### Actin staining

Cells were incubated with fullerenol nanoparticles (200 μg/mL) in glass bottom dish for 24 h. The cells were washed with PBS, fixed with 4% paraformaldehyde for 15 min and then punched with 0.1% Triton X-100 for 15 min. The F-actin cytoskeleton was stained with Rhodamine-labeled phalloidin (Cytoskeleton, USA) for 20 min at room temperature, and the nuclear was counterstained with Hoechst 33342 (Thermal fisher scientific, USA) for 20 min. Images were acquired using Laser Scanning Confocal Microscope.

### Western blot

G-actin (soluble actin) and F-actin (insoluble actin) were extracted according to the method described previously [20]. We prepared soluble actin extraction solution (50 mM Tris–HCl, 300 mM Sucrose, 25 mM NaCl, 2 mM EGTA, 25 mM NaF, 0.2% Triton X-100, 1 mM NaCO_3_, 1 mM PMSF, and protease inhibitor cocktail) and insoluble actin extraction solution (RIPA Lysis Buffer (Beyotime, China), 1 mM PMSF and protease inhibitor cocktail). After treatment various concentration of fullerenol nanoparticles for 24 h, the cells were lysed for 5 min at 4 °C by soluble actin extraction solution. G-actin solution was collected from lysates supernatant. Then, the cells residual was lysed in RIPA Lysis Buffer containing PMSF for 2 h at 4 °C, F-actin solution was gathered. Proteins were separated by SDS PAGE gel, and transferred onto immobilon^®^- P transfer membrane. Membrane were blocked with 5% skim milk for 1 h and probed with anti-ACTIN, anti-GAPDH (TransGen Biotech, China) overnight at 4 °C. Finally, second HRP-conjugated antibodies (ZSGQ, China) incubated 2 h at room temperature. The result was acquired using automated chemiluminescence imaging analysis system (Tanon 5200, China). Grayscale analysis performed by Image J.

### Actin polymerization in vitro

G-actin powder (Cytoskeleton, USA) was dissolved in G-actin buffer (5 mM Tris–HCl, 0.2 mM CaCl_2_, 0.2 mM ATP, 1 mM DTT) to give a final concentration of 0.5 mg/mL G-actin solution. The G-actin solution was incubated with or without 1 μg/mL fullerenol nanoparticles for 1 h at 4 °C. Polymerization-Buffer (P-buffer: 500 mM KCl, 20 mM MgCl_2_.6H_2_O, 10 mM ATP-2Na) was added and incubated for 30 min at room temperature. 200 μL mixture of G-buffer and P-buffer (1:1) were added before being labeled with Rhodamine-labeled phalloidin. The samples dried at room temperature and imaged by laser Scanning Confocal Microscope.

### Detection of Young’s modulus

The cell suspension of MCF-7, MDA-MB-231 cells and MCF-10A cells (5 × 10^5^ cells/well) were seeded in glass dish. After 24 h, the cells were incubated with fullerenol for 24 h, Basal medium softly washed with three times. Young’s modulus was detected by atomic force microscope. We used the HQ: NSC15/ALBS probes with a nominal spring constant of K = 40 N/m, from Mikromasch probes.

### Scratch wound-healing assay

Scratch wound-healing was performed to detect the ability of cell migration. The cells were cultured on 6-well plate for 24 h, and then scratched with a 200 μL pipette tip after cells attached. The cells washed with PBS three times to avoid detached cells, and then replaced with completed medium in the absence or presence of fullerenol. 0, 24 h after wounding, images were acquired by fluorescent inverted microscope. The cell migration rate of control was considered as 100% and the healing rate of other plates were compared to control cells.

### Transwell migration assay and invasion assay

Transwell plate with 24-well, 8.0 μm pore size Transparent PET Membrance (Corning, USA) was used for cell migration and invasion assays. The upper chambers were seeded with 0.5 mL cell suspension and the bottom chambers were added to 0.8 mL completed medium. After the cells were attached, the completed medium with proper concentration fullerenol was treated.

For the transwell invasion assay, the insert membrane was coated with 80 μL Matrigel (BD Biosciences, USA) (20 μL Matrigel and 60 μL L15 serum-free medium) and dried for 1 h in cell incubator. Then, the 100 μL cell suspension with proper concentration fullerenol in serum-free medium (5 × 10^4^ cells/well) was added into the upper chamber, and the lower chamber was added with medium containing 10% FBS as chemoattractant. After 24 h of incubation, the upper chambers were fixed with 4% paraformaldehyde for 15 min. The Matrigel and upper cells were wiped with cotton balls. The lower migrated cells stained with crystal violet for 30 min, and counted migrated cells in five random microscopic fields using microscope.

### Immunofluorescence of integrin

The cells were cultured in sterile glass bottom dish, and 200 μg/mL fullerenol nanoparticles was treated for 24 h. The cells washed with PBS three times and fixed with 4% paraformaldehyde for 20 min at room temperature. Then, it was incubated in 1% BSA/10% normal goat serum/0.3 M glycine in 0.1% PBS-Tween for 2 h at room temperature to permeabilise and block the non-specific protein–protein interactions. PBS washed three times (15 min/times). The cells were incubated with Anti-integrin (ab30394 at 10 μg/mL, Abcam UK) for 2 h at room temperature, and PBS washed three times (15 min/times). The secondary antibody was a Goat Anti-Mouse Alexa Fluor 488 (lgG H&L) (ab150117, Abcam UK) used at 1/200 dilution for 2 h at room temperature. The F-actin cytoskeleton was labeled with Rhodamine-labeled phalloidin for 20 min at room temperature, and Hoechst 33342 was used to stained the cell nuclei for 20 min. Fluorescent images were obtained from laser Scanning Confocal Microscope.

### Determination of integrin expression by flow cytometry

The cell suspension with different treatment was centrifuged and collected, then the cells were fixed with 4% paraformaldehyde for 20 min. 1% BSA/10% normal goat serum/0.3 M glycine in 0.1% PBS-Tween was added to block non-specific binding sites. The cells were incubated with Anti-integrin (1 μg/mL) and the secondary antibody at 1:200 dilution for 30 min at 22 °C, respectively. Acquisition of > 5000 events was performed by flow cytometry.

### Scanning electron microscope to determine cell morphology

The cells were seeded on small wafer overnight at 37 °C with 5% CO_2_, and treated 200 μg/mL fullerenol for 24 h. Next day, the cells washed with PBS three times and fixed with 2.5% glutaraldehyde for 20 min. PBS and sterile water washed three times, respectively. The samples were dehydrated followed by 30, 50, 70, 90% ethanol for 10 min and dried at room temperature overnight. Cell morphology were observed by Field Emission Scanning Electron Microscope (JSM-6701F, Japan). We randomly selected 50 cells from the control and fullerenol group, respectively. The number of filopodia longer than 1 μm were counted.

### Establishment of lung metastasis model and antimetastatic therapy in vivo

Five-week-old female nude mice were randomly divided into three groups (control, fullerenol, and blank group, n = 5/group). Body weights of nude mice were recorded every alternate day. The MDA-MB-231-luc cells were injected into mice at a density of 1 × 10^6^ cells/mouse via tail vein to develop a lung metastasis model of breast cancer in control group and fullerenol group. Blank group were injected saline. Seven days after injection, the mice were injected fullerenol solution (35 mg/kg body weight) or saline daily for 5 weeks by tail intravenous injection. After 14 days of injection, the development of a lung metastasis model was predetermined under IVIS Spectrum imaging system (PerkinElmer, USA). The mice were treated with 150 mg/kg of d-luciferase (Aladdin, USA) by intraperitoneal injection at different days (21, 28, and 35). At 10–15 min after injection, the signal of model mice was monitored by IVIS Spectrum imaging system. In order to further determine the metastatic foci, at 42 days, the mice were sacrificed, and the major organs were imaged by the IVIS Spectrum. Besides, mouse organs were fixed with 4% paraformaldehyde, sectioned, and stained with hematoxylin and eosin.

### Immunohistochemistry (IHC) staining

Lung tissue slices of model mouse in different groups were labeled by immunohistochemistry (IHC) staining. Vimentin, a specifical label of human MDA-MB-231 cells, was selected to mark cancer cells in lung tissue [21, 22]. The sections were dewaxed in xylene, and dehydrated in an alcohol gradient of 100, 95, 75% for 10 min. Then, heat mediated antigen retrieval was performed using citrate buffer (pH 6.0). Endogenous peroxidase activity was blocked by 3% H_2_O_2_ for 10 min. Next, 3% BSA/0.3% Triton X-100 was used to permeabilise and block nonspecific binding, followed by incubation with the anti-Vimentin antibody (ab92547, 1:200) at 4 °C overnight in a moist chamber. After washing three times with PBS, the slices were incubated with a secondary antibodies (ZSGQ, China) at room temperature for 1 h and washed with PBS three times. Diaminobenzidine (DAB) was used as the chromogen for 5 min-10 min. Finally, the sections were counterstained with hematoxylin. No primary antibody was used in the negative control.

### Statistical analysis

The analysis of differences between the groups were evaluated by SPSS software, statistical difference at P < 0.05 was defined as significant differences and P < 0.01 was considered very significant differences.

## Results

### Characterization and cytotoxicity assessment of fullerenol

**Fig. 1:**
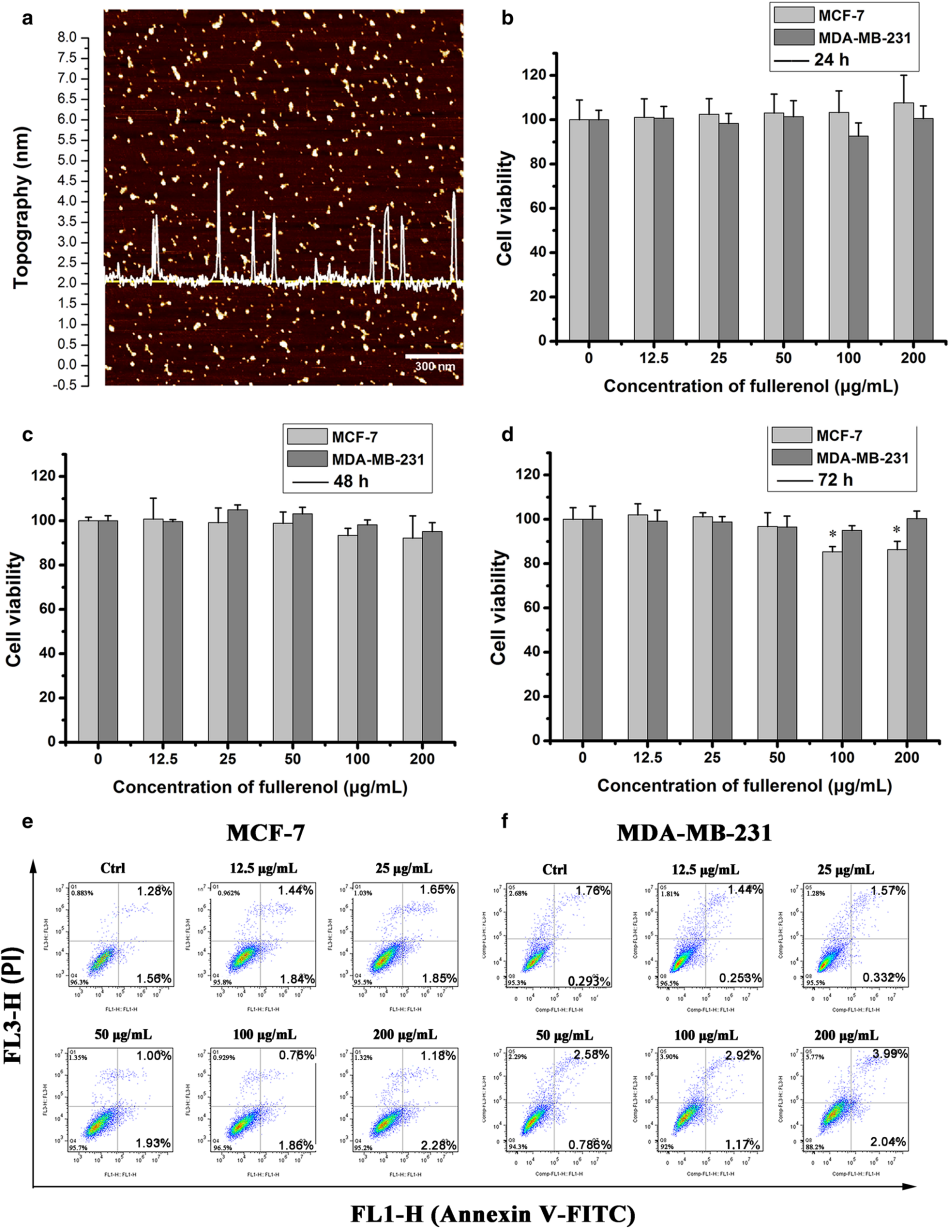
Fullerenol nanoparticle characterization and cytotoxicity assessment. **a** Typical image of AFM. **b** The influence of fullerenol on breast cancer cell viability. MCF-7 and MDA-MB-231 cells were incubated with fullerenol (12.5, 25, 50,100, or 200 μg/mL) for 24 h (B), 48 h (**c**), 72 h (**d**) and cell viability was detected by CCK-8. Error bars represent mean ± SD, *P < 0.05 and **P < 0.01 (n = 6). Apoptosis induced by fullerenol in breast cancer cells. Cells were stained with Annexin V-FITC and PI for 30 min. Flow cytometry analyzed apoptosis in MCF-7 (**e**) and MDA-MB-231 cells (**f**) treated with fullerenol for 24 h

Hydrosoluble fullerenol nanoparticles were separated and purified as our previous work [14]. AFM was used to detect diameter of these fullerenol nanoparticles. As shown in Fig. 1a, the size of fullerenol nanoparticles distributed on the yellow line is presented by waveforms, and the diameter of 100 μg/mL fullerenol in PBS was 1.72 ± 0.14 nm. Dynamic light scattering (DLS) was used to detect the zeta potential of fullerenol particles, which was determined to be − 17 mV in PBS. Fullerenol from 12.5 to 200 µg/mL did not significantly affect the viability of breast cancer cells (MCF-7 and MDA-MB-231 lines) with the prolonged treatment time (Fig. 1b–d). Live/dead cell assays confirmed that the fullerenol did not appreciably induce cell death (Additional file 1: Figure S1).

Cancer cell apoptosis was measured with annexin IV-FITC/PI apoptosis detection kits. Fullerenol treatment (from 12.5 to 200 µg/mL) did not obviously increase the apoptosis rate of MCF-7 or MDA-MB-231 cells in contrast with control (Fig. 1e, f). Decreased mitochondrial membrane potential is a marker of early apoptosis, and fullerenol reportedly affects the mitochondrial membrane potential in kidney cells [23]. JC-1 was therefore utilized to evaluate early apoptosis. Red JC-1 aggregates indicate healthy mitochondria, whereas green JC-1 monomer is observed in the setting of mitochondria membrane damage. Fluorescent images show that fullerenol did not induce observably increase green JC-1 monomers in MCF-7 and MDA-MB-231 cells (Additional file 1: Figure S2). This indicates that fullerenol does not induce apoptosis by altering mitochondrial membrane potential in MCF-7 or MDA-MB-231 cells.

### Fullerenol inhibits breast cancer cells to found metastasizing colonies in the lung

**Fig. 2:**
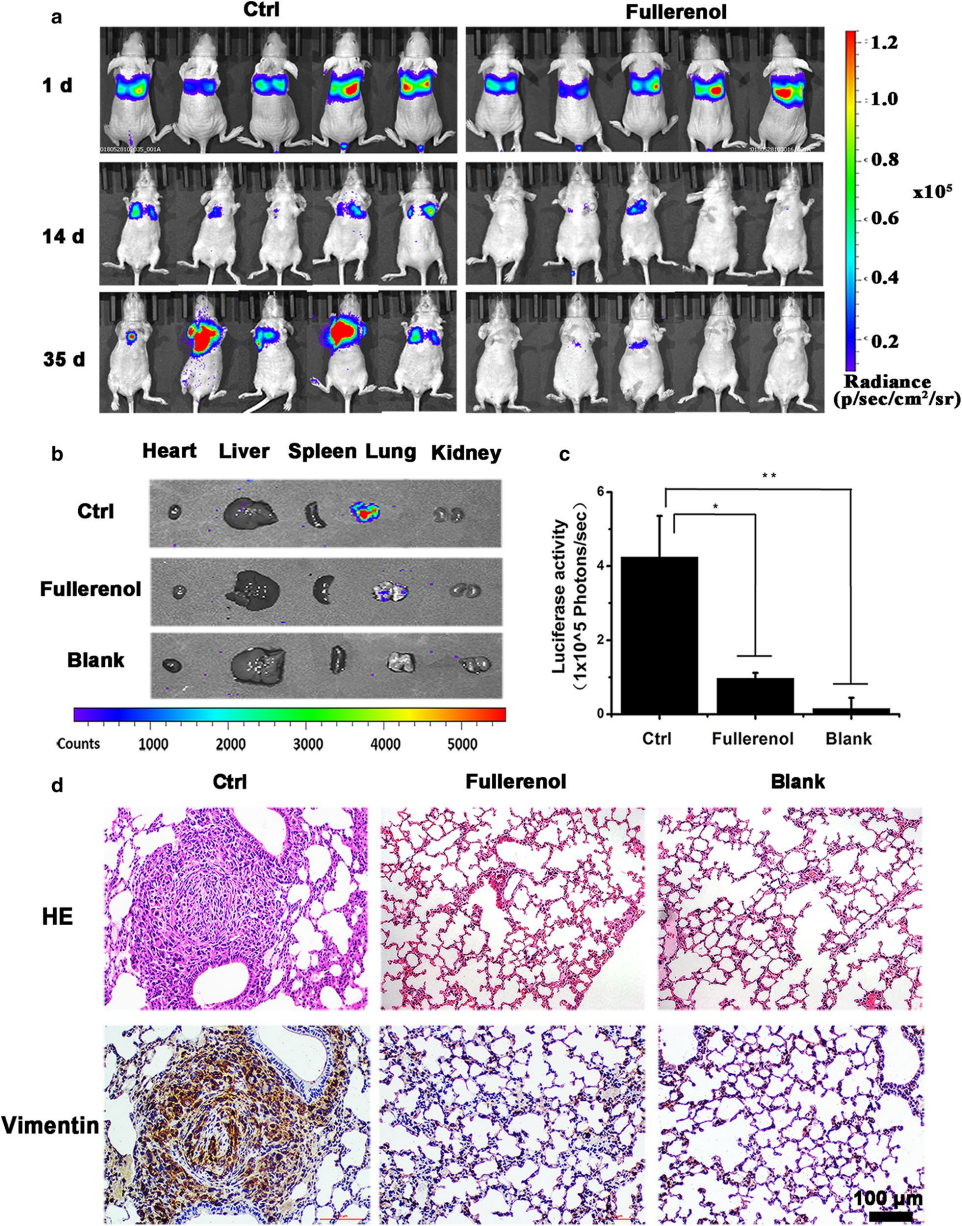
The therapeutic efficacy of fullerenol nanoparticles to prevent lung metastasis. **a** Bioluminescence images of mice after injection of breast cancer cells on days 1, 14 and 35. **b** Metastatic foci distribution in nude mice. Typical ex vivo images of major organs from each group at 42 days after injection. Fluorescence intensity was detected with a spectrum/CT multimodal imaging system. **c** Quantified lung distribution in each group, *P < 0.05, **P < 0.01, n = 5. **d** H/E and vimentin staining of lung tissue section from different groups, scale bar = 100 μm

The inhibitory effect of fullerenol on metastasis of breast cancer cells through the vasculature was evaluated with an IVIS Spectrum imaging system. Five-week-old female nude mice were randomly divided into three groups (control, fullerenol, and blank group, n = 5/group), and MDA-MB-231 cells with stable luciferase expression were intravenously delivered via the caudal vein. We performed bioluminescence imaging of mice in the first 24 h after the injection of the cells and found strong fluorescence in the lung, indicating that the breast cancer cells injected intravenously have accumulated in the lung (Fig. 2a). After 7 days, fullerenol solution (35 mg/kg body weight) or saline was intravenously injected daily for 5 weeks. The dose was ascertained depending on our preliminary experiment of acute toxicity test (data was not shown). Body weights were recorded every other day and were not significantly different among the three groups (Additional file 1: Figure S3). Metastatic colonies of MDA-MB-231 cells in modeling mouse were detected by bioluminescence assays. Metastatic colonies with blue fluorescence appeared in the lungs of all mice in the control group (5/5), compared to only one mouse in the fullerenol group (1/5). From day 14 to day 35, the intensity and distribution of the labeled breast cancer cells increased in the lungs of control mice (Fig. 2a). Interestingly, fluorescence intensity was reduced in the lung of fullerenol-treated mice. At 42 days, the mice were sacrificed, and the major organs were imaged by the IVIS Spectrum (Fig. 2b). The fluorescence intensities in lung of mouse were significantly different in the control and treated groups (539 × 10^5^ p/s and 3.7 × 10^5^ p/s, respectively; Fig. 2c), and did not observed in other organs.

Mouse organs were fixed with 4% paraformaldehyde, sectioned, and stained with hematoxylin and eosin. In other organs except the lung, there were no colonies of breast cancer cells (Additional file 1: Figure S4). Conversely, colonies appeared in the lungs of all mice in control group, but were only rarely visualized in the treated group (Fig. 2d, Additional file 1: Figure S5). We used the antibodies of anti-vimentin to detect the cancer cells in lung tissue of all groups. In the control group, positive labels of vimentin present in lung and indicate formation of micrometastatic colonies (Fig. 2d). In lung tissue slices of treatment group, the positive labels was not detected. This indicates that fullerenols prevent breast cancer cells from founding tumor colonies in the lung.

### Fullerenol nanoparticles disturb actin cytoskeleton assembly

**Fig. 3:**
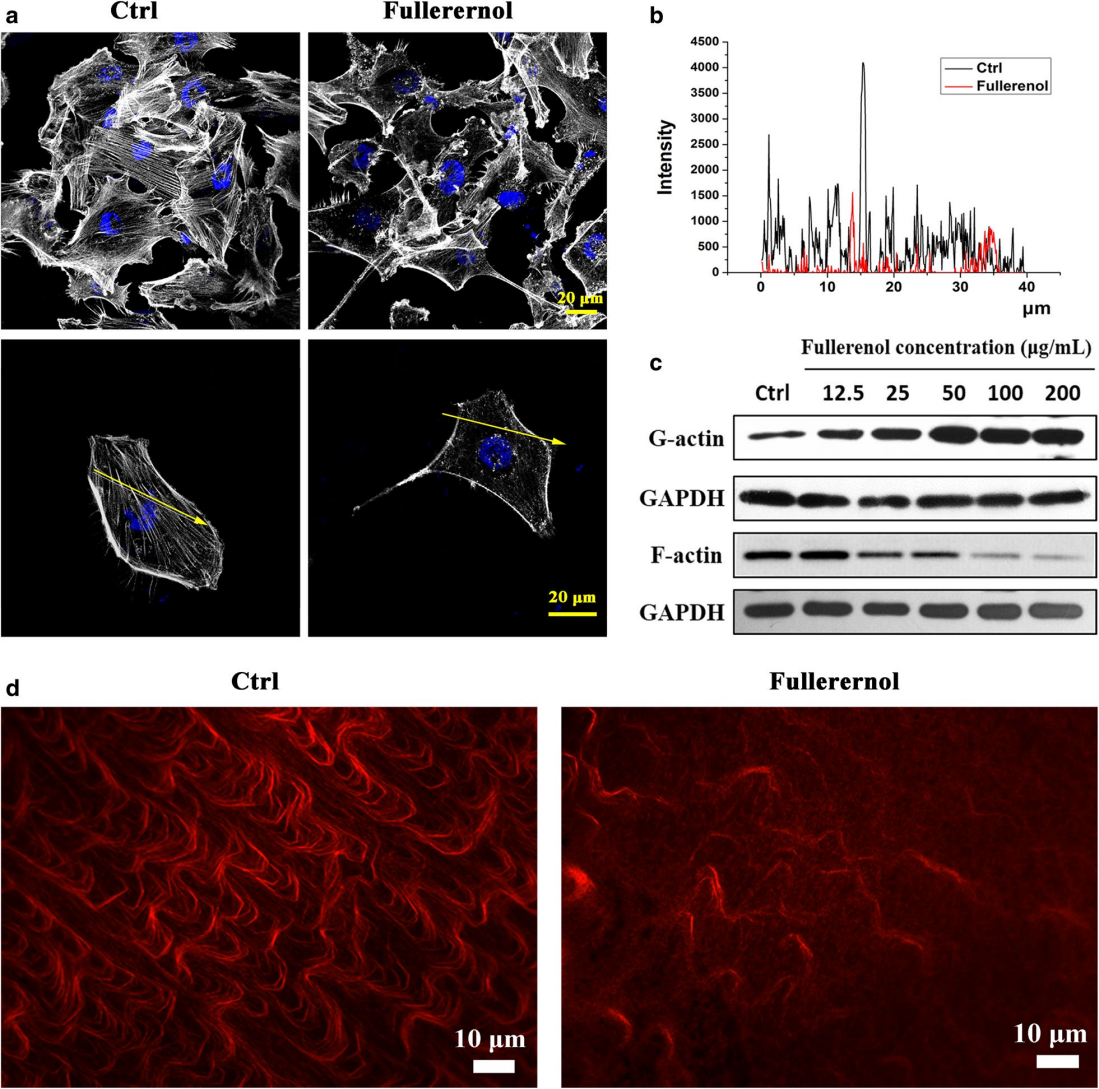
Effect of fullerenol nanoparticles on the actin cytoskeleton. **a** Actin filaments were clearly visible and orderly in control MDA-MB-231 cells, while fullerenol-treated cells had disrupted and reduced actin fibers. Scale bar = 20 μm. The fluorescence intensity of labeled F-actin obtained by laser scanning confocal microscopy. **b** Western blots of G-actin and F-actin expression in MDA-MB-231 cells. **c** In *vitro* actin polymerization assays. In control cells, the actin fibers were tight and ordered, but fullerenol treatment significantly reduced the number of actin fibers, which were also thinner and shorter. Scale bar = 10 μm

Dynamic reorganization of the cytoskeleton leads to cell deformability that allows migration and invasion [24–27]. Here we confirmed that fullerenol influenced actin cytoskeleton reorganization in cancer cells. Control cells exhibited straight, strong, well-arranged actin fibers, but disordered arrangement of thin actin fibers was evident in treated cells with 200 μg/mL fullerenol (Fig. 3a). The fluorescence intensity of labeled actin was lower in treated cells, and the amount of actin fiber was obviously reduced compared with control (Fig. 3b). Actin cytoskeleton reorganization was also observed in other treated cancer cell lines including MCF-7, Caco-2, HeLa, HepG-2, MB-49 cells and non-tumoural cell line such as MCF-10A (Additional file 1: Figure S6).

Western blots were performed to assay G-actin and F-actin levels. Fullerenol dose-dependently (12.5, 25, 50, 100, and 200 µg/mL) reduced and increased the contents of F-actin and G-actin, respectively (Fig. 3c). Similar results were observed in MCF-7 cells (Additional file 1: Figure S7). This result demonstrates that fullerenol alters the dynamic balance of F-actin and G-actin in cancer cells.

The interference of fullerenol with actin assembly was also shown by in vitro actin polymerization assays. Actin fibers were clearly and visibly arranged in control but were diffused in treatment (Fig. 3d). This indicates that fullerenol regulates the assembly of G-actin into F-actin and disturbs actin cytoskeleton reorganization.

### Disrupted actin dynamics affects the Young’s modulus of cancer cells

**Fig. 4:**
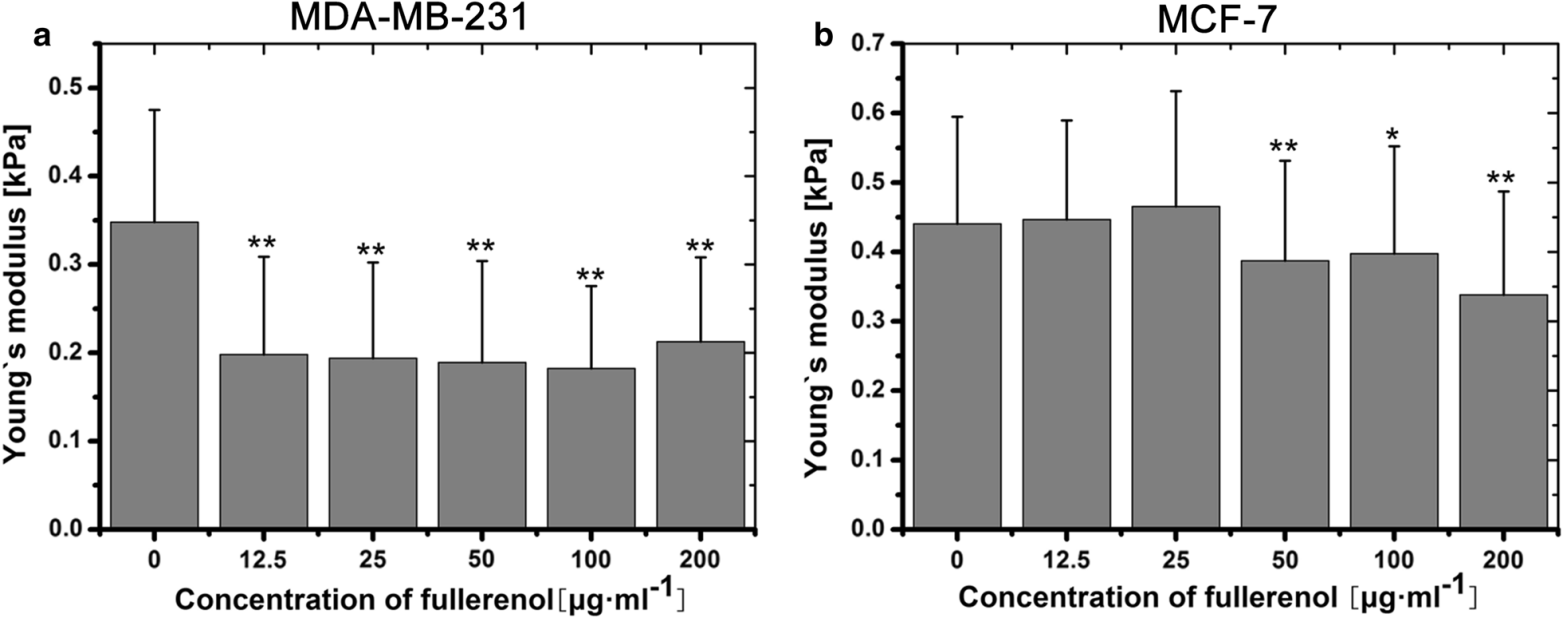
The evaluation of cell’s stiffness. Young’s modulus values obtained by AFM to assess the stiffness of MDA-MB-231 cells (**a**) and MCF-7 cells (**b**). The cells were treated with fullerenol for 24 h. Error bars represent mean ± SD; *P < 0.05 and **P < 0.01 (n ≥ 100)

Dynamic cytoskeletal reorganization regulates cellular biomechanical properties such as migration, adhesion and even metastasis [6, 26, 28]. To achieve metastasis, malignant cells must be able to deform by remodeling the actin cytoskeleton [29–32]. Variable cellular stiffness is a typical property of malignant tumor [33, 34]. We performed AFM to measure the Young’s modulus values of breast cancer cells (MDA-MB-231 and MCF-7 cells) and normal cells (MCF-10A cells). Compared with control cells, the Young’s modulus of fullerenol-treated MDA-MB-231 cells were obviously different. Fullerenol (from 12.5 to 200 μg/mL) significantly decreased the Young’s modulus values of MDA-MB-231 cells and MCF-10A cells (Additional file 1: Figure S8), and above 50 μg/mL significantly impacted MCF-7 cells’ values (Fig. 4a, b). This indicated that fullerenol decreases cell stiffness.

### Disordered actin cytoskeleton inhibits filopodia formation at polar locations and redistribute integrin in breast cancer cells

**Fig. 5:**
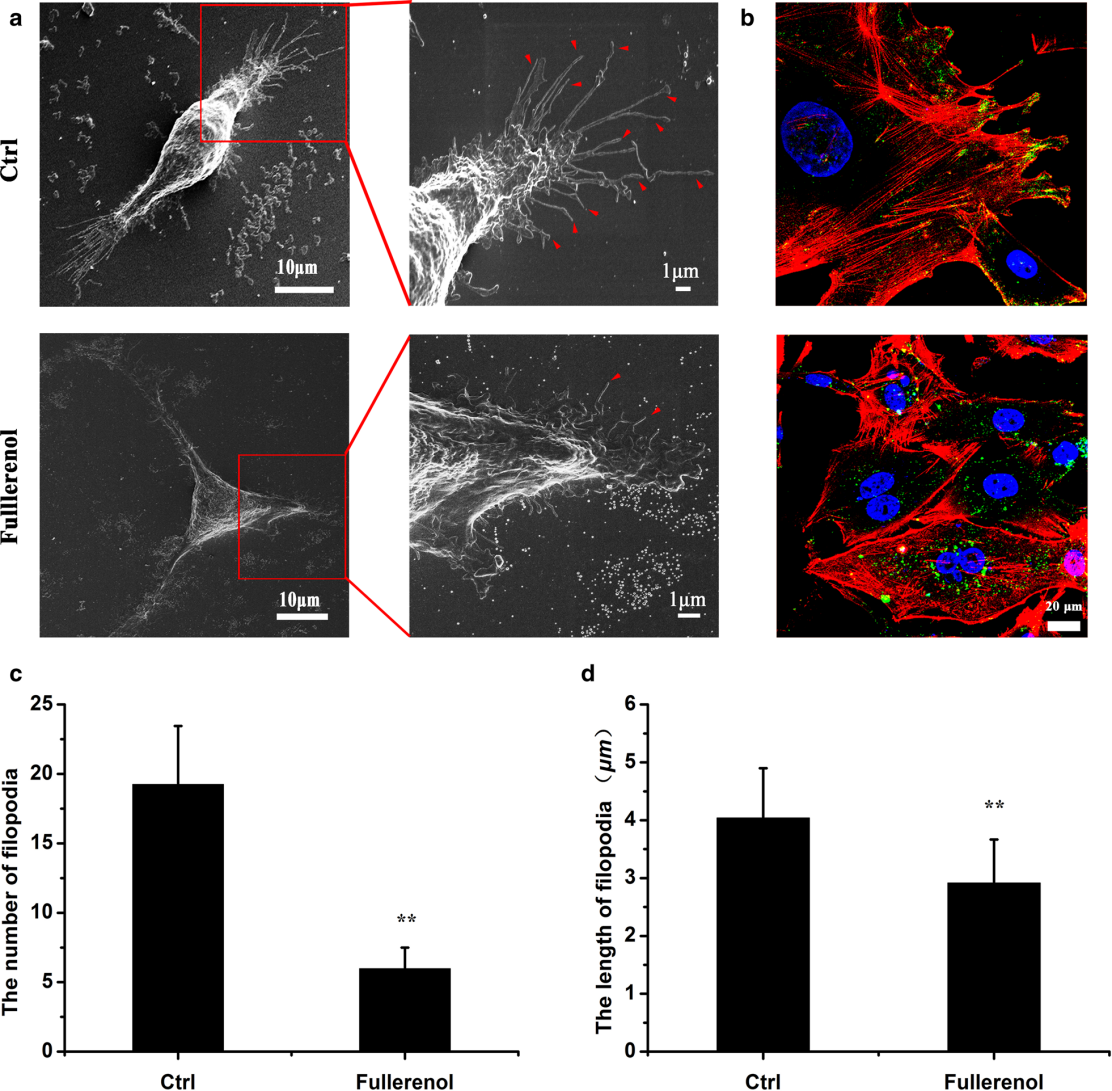
The influence of fullerenol on filopodia formation and integrin distribution. **a** SEM image of MDA-MB-231 cells. Control cells or those treated with 200 μg/mL fullerenol nanoparticles for 24 h were fixed and dehydrated. Control cells showed numerous spindly protrusions, whereas treated cells displayed short protrusions. **b** Immunofluorescence images of phalloidin staining in MDA-MB-231 cells. Green = integrin β1, red = actin cytoskeleton, blue = nucleus. Scare bar = 20 μm. **c**, **d** A quantification for the number and length of filopodia. n ≥ 50, *P < 0.05, **P < 0.01

Filopodia are actin-rich protrusions that facilitate cancer cell motility and invasion [35–37]. We monitored filopodia ultrastructure under SEM and found abundant, spindly filopodia in polar locations of control cell, but short and crooked filopodia in treated cell (Fig. 5a). Moreover, the number of filopodia longer than 1 μm was counted under SEM. After treatment of 200 μg/mL fullerenol, the average number of filopodia decreases from 19 to 6/per cell, and length of filopodia shortens from 4.04 to 2.92 μm (Fig. 5c, d). It indicated that fullerenol could significantly decrease the number and length of filopodia. The primary support structures of filopodia are actin bundles, and reduced F-actin levels could explain the disappearance and variation of filopodia in cancer cells.

Integrins are the main extracellular matrix (ECM) receptors that link the ECM with the intracellular cytoskeleton and control cell proliferation and movement [38]. Integrin clusters are useful biomarkers of cell adhesion because they correlate with actin organization and tumor metastasis [39–41]. To determine whether actin cytoskeleton reorganization affects integrin distribution in fullerenol-treated cells, immunofluorescence assays were performed to label integrin in breast cancer cells. Green-labeled integrin was mainly distributed in filopodia in control cells, whereas it was largely found in the cytoplasm of treated cells (Fig. 5b, Additional file 1: Figure S9). Flow cytometry was performed to evaluate the fluorescence signal of integrin in fixed breast cancer cells; there was no obvious difference between treated and control cells (Additional file 1: Figure S10). This suggests that fullerenol disturbs actin cytoskeleton reorganization and alters the intracellular distribution of integrin, which could explain the low adhesion ability of treated.

### Cell migration and invasion is correlated with the decrease of actin fiber

**Fig. 6:**
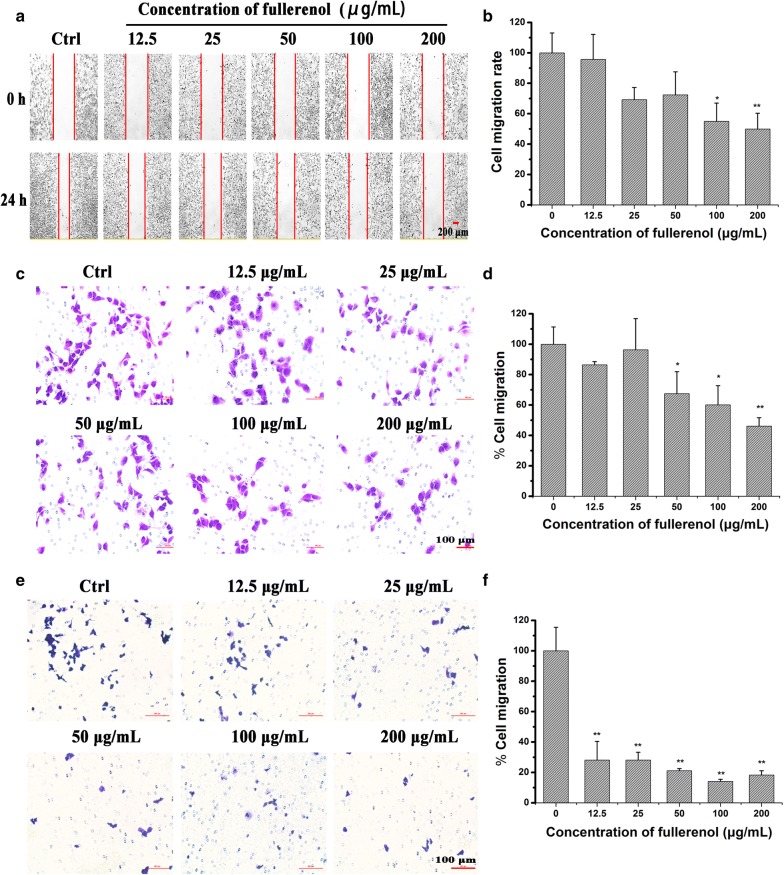
In *vitro* inhibitory effects of fullerenol nanoparticles on cell migration. Typical images of scratch wound-healing assays (**a**), Transwell migration assay (**c**), and Transwell invasion assays (**e**) performed in MDA-MB-231 cells. The statistical results of scratch wound-healing assays (**b**), Transwell migration (**d**) and invasion assays (**f**). Scale bar = 200 μm in (**a**), and scale bar = 100 μm in (**c**) and (**e**). The inhibitory effect was normalized to control values, *P < 0.05, **P < 0.01

Scratch wound-healing assays were used to evaluate cell migration, and fullerenol significantly restrained MDA-MB-231 cell migration at 200 μg/mL compared with control (Fig. 6a, b). Fullerenol similarly inhibited the migration of other cancer cell types (Additional file 1: Figure S11).

We also elucidated the role of fullerenol in inhibiting breast cancer cell migration using transwell migration assay. Compared with control cells, ~ 54% MDA-MB-231 cells were arrested by 200 μg/mL fullerenol (Fig. 6c, d). Next, we evaluated the anti-invasion property of fullerenol using transwell invasion assay. The membranes of upper chambers were coated with Matrigel, and the numbers of cells that crossed and then attached to the back of membrane were counted. Result of the assay performed with MDA-MB-231 cells highlighted that fullerenol at 12.5–200 μg/mL significantly reduced the number of invasive cancer cells. Even at a fullerenol concentration of 12.5 μg/mL, only 28% of cells were able to invade and cross the membrane coated Matrigel (Fig. 6e, f). This result indicates that fullerenol restrains MDA-MB-231 cell migration and invasion.

## Discussion and conclusion

Migratory breast cancer cells in lymphatic and blood vessels seek new sites and are more likely to form metastatic colonies in lung and bone [42, 43]. Once they establish themselves in distant tissues, these cancer cells often wreak considerable havoc. As such, these wandering cells are danger manufacturers in cancer development. Our results show that fullerenol nanoparticles can markedly inhibit the founding of metastatic breast cancer cell colonies in the lung (Fig. 2).

Potentially metastatic cells, which undergo complex deformation to execute a series of complex physiology processes including adhesion, migration, invasion, acquire the ability to form new colony in distant organization [44]. Continuous actin cytoskeleton reorganization is a critical prerequisite for cancer cell deformation [45, 46]. We investigated the effect of fullerenol on G-actin assembling into F-actin and showed increased G-actin content and obviously less F-actin in fullerenol treated cells (Fig. 3). The result indicated that fullerenol interferes with the physiological equilibrium of G-actin and F-actin, thereby disrupting the cytoskeleton. Interestingly, the variation induced by fullerenol did not reduce cell viability or enhance apoptosis (Fig. 1).

Previous studies described a close relationship between cancer cell stiffness and metastasis [45, 47], and softened cells are much more invasive [33, 34]. We found that the Young’s modulus value of cancer cells was decreased by fullerenol, but their motility and invasive capabilities were also weakened, thus inhibiting metastasis to the lung (Figs. 2, 4). This indicates that there is a cell physiological stiffness threshold for motility. Once the threshold is exceeded, cancer cells cannot adjust cytoskeletal dynamics to form new functional structures to facilitate invasion and metastasis. Fullerenol nanoparticles were likely to broken the threshold and lead breast cancer cell to lose the capacity of dynamically regulating actin cytoskeleton reorganization for cell migration and invasion.

In migrating cancer cells, spike-like filopodia are supported by the reorganization of actin fibers that are tightly bundled beneath the plasma membrane of each structure [48]. SEM showed that fullerenol reduced the number and length of filopodia, leaving them unable to attach to the ECM, whereas strong and powerful filopodia were observed in control cells (Fig. 5a). Fullerenol visibly disturbed actin fiber assembly, and the number of actin fibers is significantly reduced compared with control (Fig. 3a, d). Therefore, formation of filopodia based on F-actin bundling was restrained and result in reduction of filopodia in fullerenol treated cancer cell.

Filopodia regulate focal contacts for cell migration and adjust the distribution of integrin at the protruding end. Integrin is a critical modulator that allows cancer cells to adhere to the ECM for founding new tumor colonies [49, 50]. Integrin expression is upregulated in various types of malignant cancers and negatively correlates with patient survival [41]. We found that integrin expression in cancer cells was not downregulated by fullerenol; rather, its distribution was altered simultaneous with cytoskeletal reorganization. Integrin distribution is polarized in control cell margin, whereas it was mainly presented in the cytoplasm of fullerenol-treated cells, and this was associated with decreased cancer cell adherence to the ECM (Fig. 5b). Since integrin is involved in the sensitization of breast cancer cells to anoikis [51, 52], we suspect that reduced colonies area in lung and varied bioluminescence images of cancer cells from 24 h to 35 day (Fig. 2a) of treated mouse may be induced by the anoikis, but this requires further study.

Our results indicate that fullerenol restrains the migration of breast cancer cells through the blood vessels and reduces the foundation of metastasis colonies in lung. This outcome is likely attributable to fullerenol’s ability to disturb dynamic actin cytoskeleton reorganization, which leads to reduced filopodia formation and altered intracellular integrin distribution, with the net effect of reduced cancer cell migration and invasiveness. Finally, lung metastasis of breast cancer via the vasculature was significantly suppressed by fullerenol. Our preliminary works support the suggestion [53] and future studies should further assess the application of fullerenols to combat metastasis.

## Additional file

**Additional file 1.** Additional figures.
