# Supplementary material for: Small size fullerenol nanoparticles suppress lung metastasis of breast cancer cell by disrupting actin dynamics
Source: J Nanobiotechnology. 2018 Jun 23;16:54. doi: 10.1186/s12951-018-0380-z (PMC6015447; doi:10.1186/s12951-018-0380-z)

Supplementary data

Small size fullerenol nanoparticles suppress lung metastasis of breast cancer cell by disrupting actin dynamics

Yanxia Qin [1, 2], Kui Chen [2,3], Weihong Gu [2, 3], Xinghua Dong [2, 3], Ruihong Lei [2], Yanan Chang [2], Xue Bai [2, 3], Shibo Xia [2, 3], Li Zeng [2], Jiaxin Zhang [2, 3], Sihan Ma [2], Juan Li [2], Shan Li [1, *] & Gengmei Xing [2, *]

[1] School of Biology and Biological Engineering, South China University of Technology, Guangzhou, 510006, People's Republic of China

[2] CAS Key Laboratory for Biomedical Effects of Nanomaterial & Nanosafety, Institute of High Energy Physics, Chinese Academy of Sciences, Beijing 100049, People's Republic of China

[3] University of Chinese Academy of Sciences, Beijing 100049, People's Republic of China

Yanxia Qin and Kui Chen contributed equally to this work

* Corresponding author

Additional figures:

Figure S1. The cytotoxicity assessment of fullerenol to breast cancer cells. Calcein-AM/PI was used to stain live and dead cells. Live cells were labeled with calcein-AM and dead cells were labeled with propidium iodide. Scale bar = 200 μm.

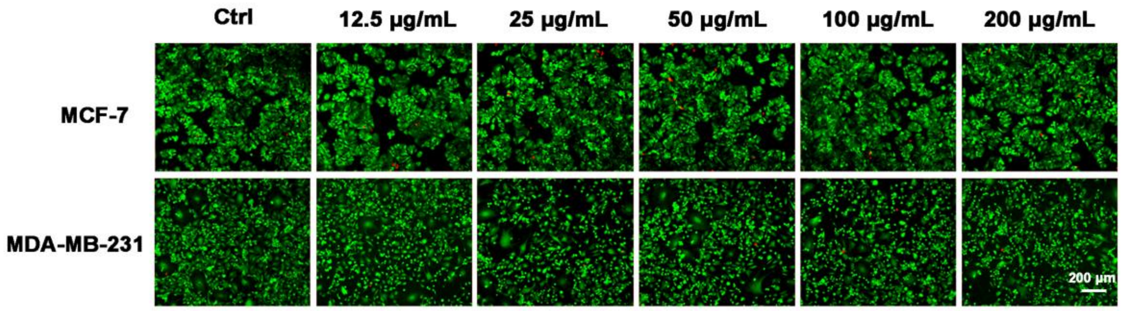

Figure S2. Early apoptosis induced by fullerenol in breast cancer cells. Staining of MCF-7 (A) and MDA-MB-231cells (B) treated with fullerenol for 24 hr with the mitochondrial membrane sensor JC-1. The red JC-1 aggregate was typical of healthy mitochondria and green JC-1 monomer represented mitochondria membrane damaged. Scale bar = 10 μm.

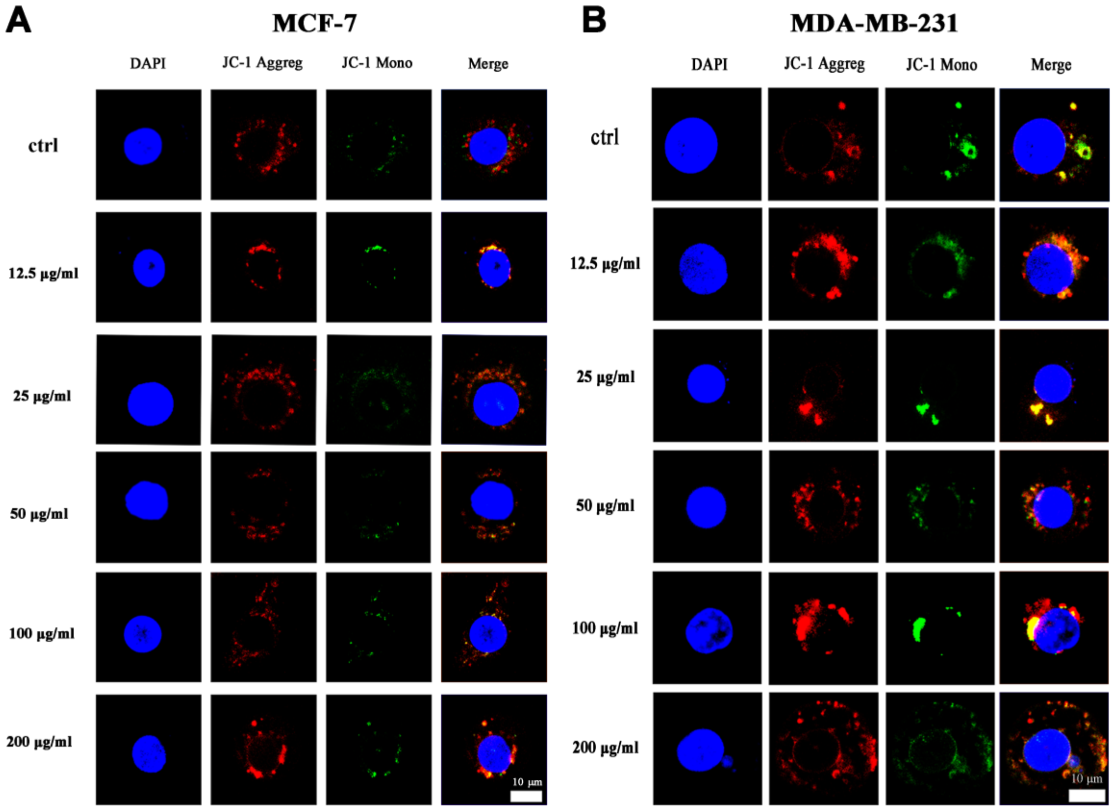
A
MCF-7
DAPI
JC-1 Aggreg
JC-1 Mono
Merge
ctrl
12.5 μg/ml
25 μg/ml
50 μg/ml
100 μg/ml
200 μg/ml
10 μm
B
MDA-MB-231
DAPI
JC-1 Aggreg
JC-1 Mono
Merge
ctrl
12.5 μg/ml
25 μg/ml
50 μg/ml
100 μg/ml
200 μg/ml
10 μm

Figure S3. Body weight-time graph. Nude mice were divided into three groups (control, fullerenol, and blank group, n=5/group). The weight of nude mice were recorded every other days. The body weight had no significant difference compared with blank group.

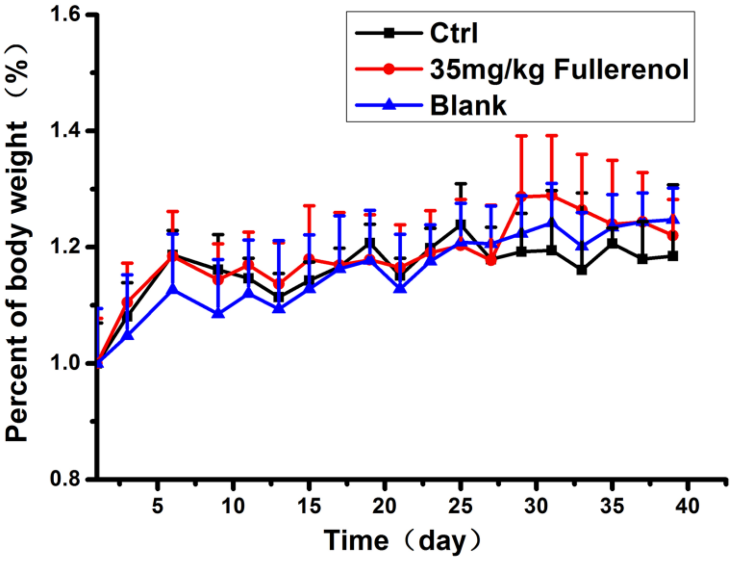

Figure S4. Histological examination of metastatic lesions in other organs (heart, liver, spleen, kidney). The metastatic microcolonies of breast cancer cell not appeared in other organs of all groups.

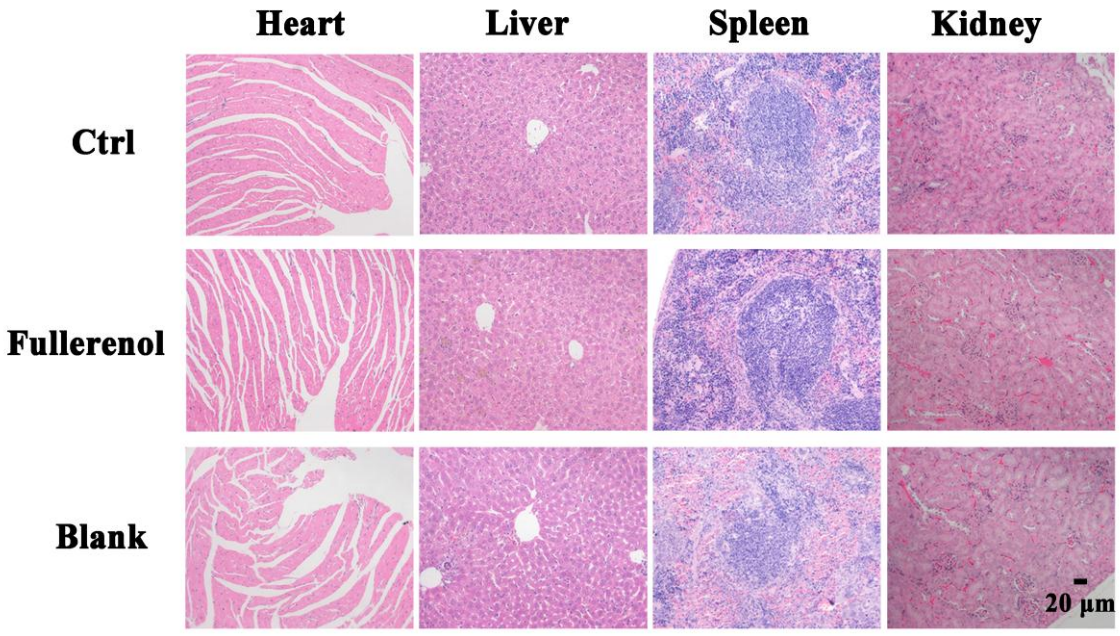

Figure S5. Histological examination of metastatic lesions in lung. The typical image of the lung tissue with different magnification and the whole lung.

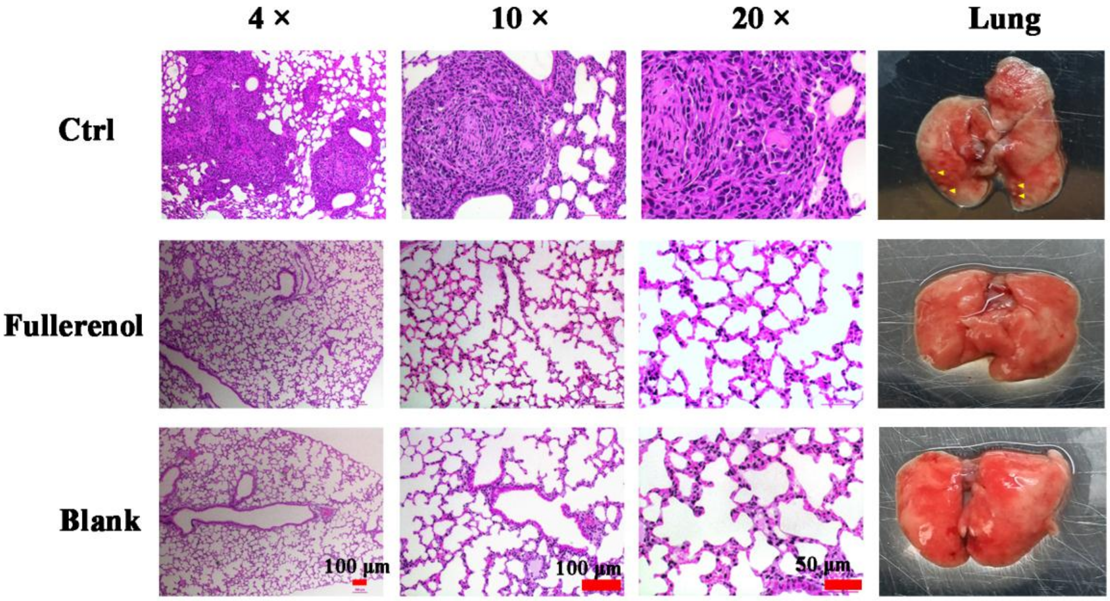

Figure S6. Effect of fullerenol on actin cytoskeleton in cancer cells. The serval cancer cell lines (A) (B) Caco-2 cells (C) (D) Hela cells (E) (F) HepG2 cells (G) (H) MB-49 cells (I) (J) MCF-7cells (K) (L) MCF-10A cells were treated with fullerenol at 200 μg/mL for 24 h. By contrast, the cells were stained with Rhodamine-labeled phalloidin and Hoechst 33342 for visualization. Scale bar = 10 μm. The actin filament was unvisible and disorder with fullerenol treatment.

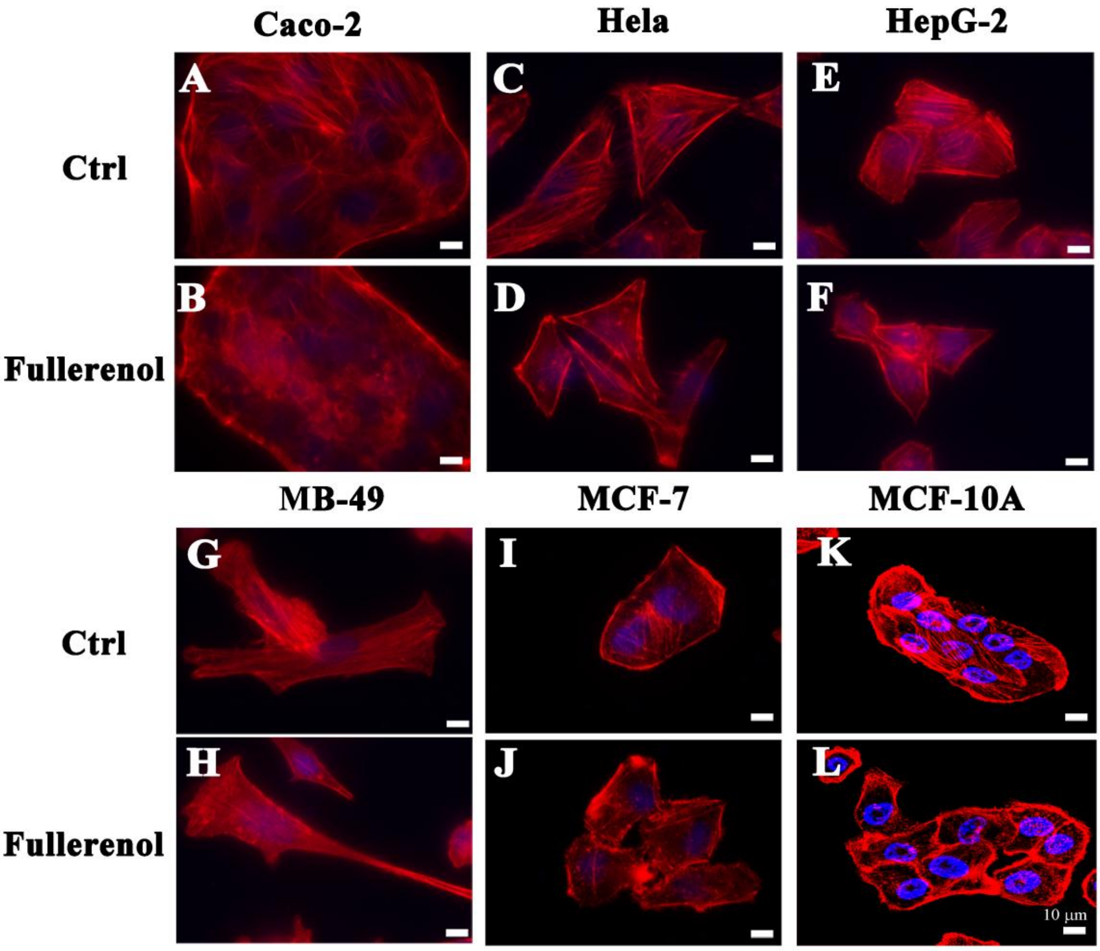

Figure S7. Western blot analyzed G-Actin and F-Actin expression in MCF-7 cells. The MCF-7 cells were treated with fullerenol at 200 μg/mL for 24 h, G-actin (soluble actin) and F-actin (insoluble actin) were extracted by soluble actin extraction solution and insoluble actin extraction solution. Fullerenol reduced content of F-actin with a dose-dependent manner and the reduction was accompanied by increased G-actin content in treated cell.

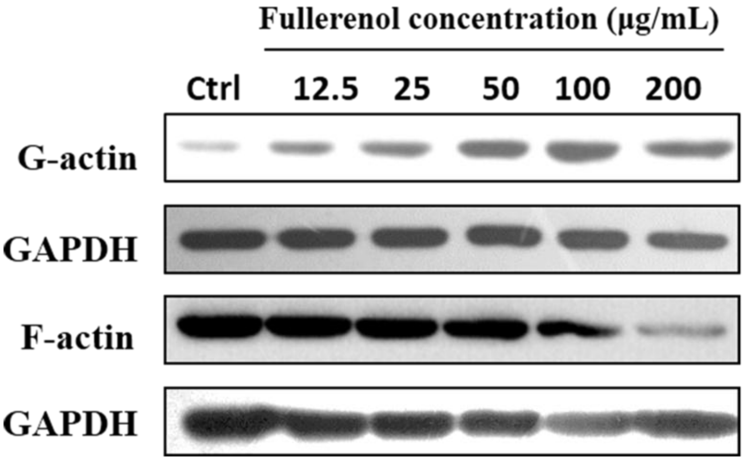

Figure S8. The evaluation of non-tumoural cell's stiffness. Young's modulus values obtained by AFM to assess the stiffness of MCF-10A cells. The cells were treated with fullerenol (200 μg/mL) for 24 h. Error bars represent mean ± SD; *P < 0.05 and **P < 0.01 (n ≥ 100).

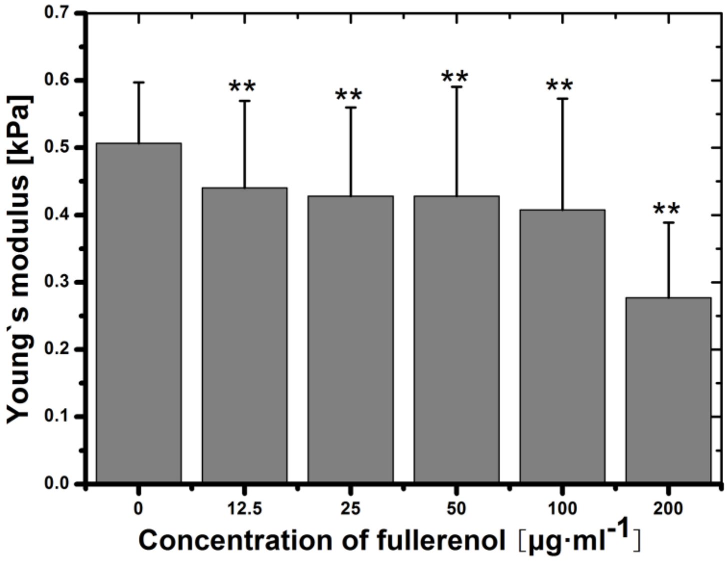

Figure S9. The influence of fullerenol on integrin β1. Immunofluorescence images of phalloidin staining in MDA-MB-231 cells treated fullerenol nanoparticles (200 μg/mL) for 24 h. Green = integrin β1, red = actin cytoskeleton, blue = nucleus. Scare bar = 20 μm.

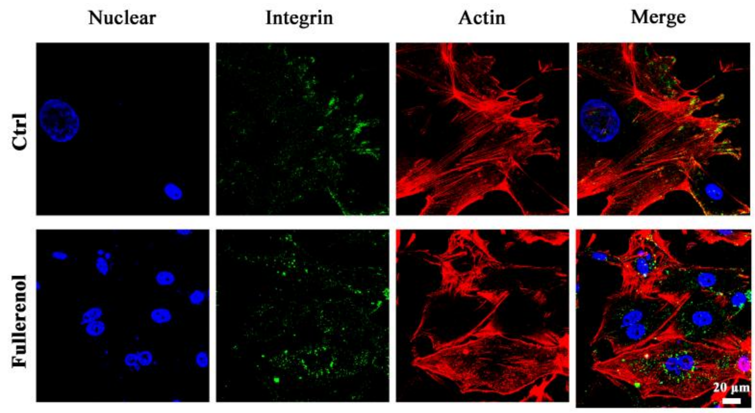

Figure S10. The evaluation of fluorescence intensity of integrin β1 by flow cytometry. The cells were cultured and 200 μg/mL fullerenol nanoparticles was treated for 24 h. Acquisition of > 5000 events was performed by flow cytometry. The content of integrin had no obvious effect on cells treated with fullerenol.

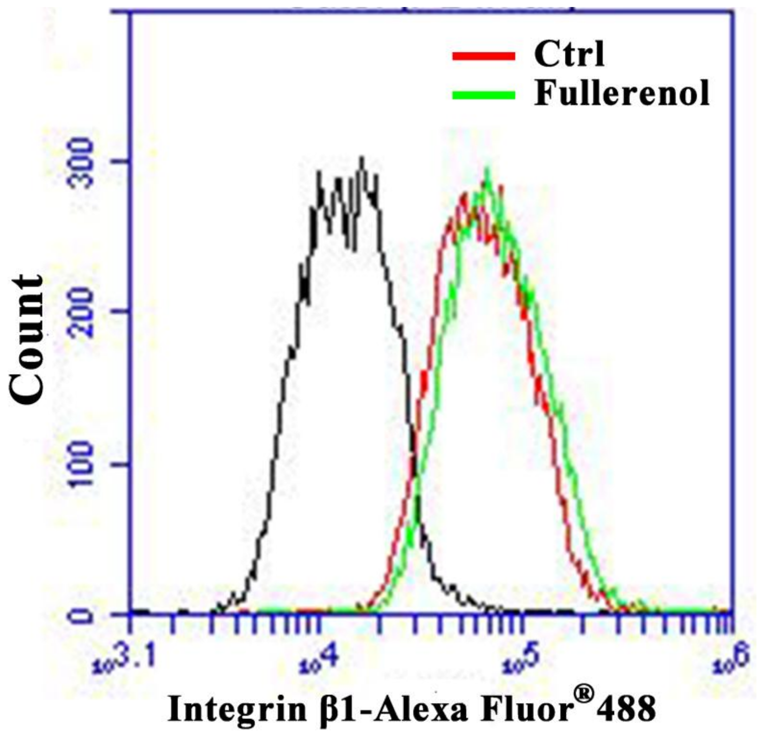

Figure S11. In *vitro* inhibitory effects of fullerenol on cell migration. The cells were treated 200 μg/mL fullerenol nanoparticles for 24 h. Would healing assay was performed to detect the

anti-migratory ability of fullerenol in (A) (B) Caco-2 cells. (C) (D) Hela cells. (E) (F) HepG-2 cells. (G) (H) MB-49 cells. (I) (J) MCF-7 cells. Scare bar = 200 μm.

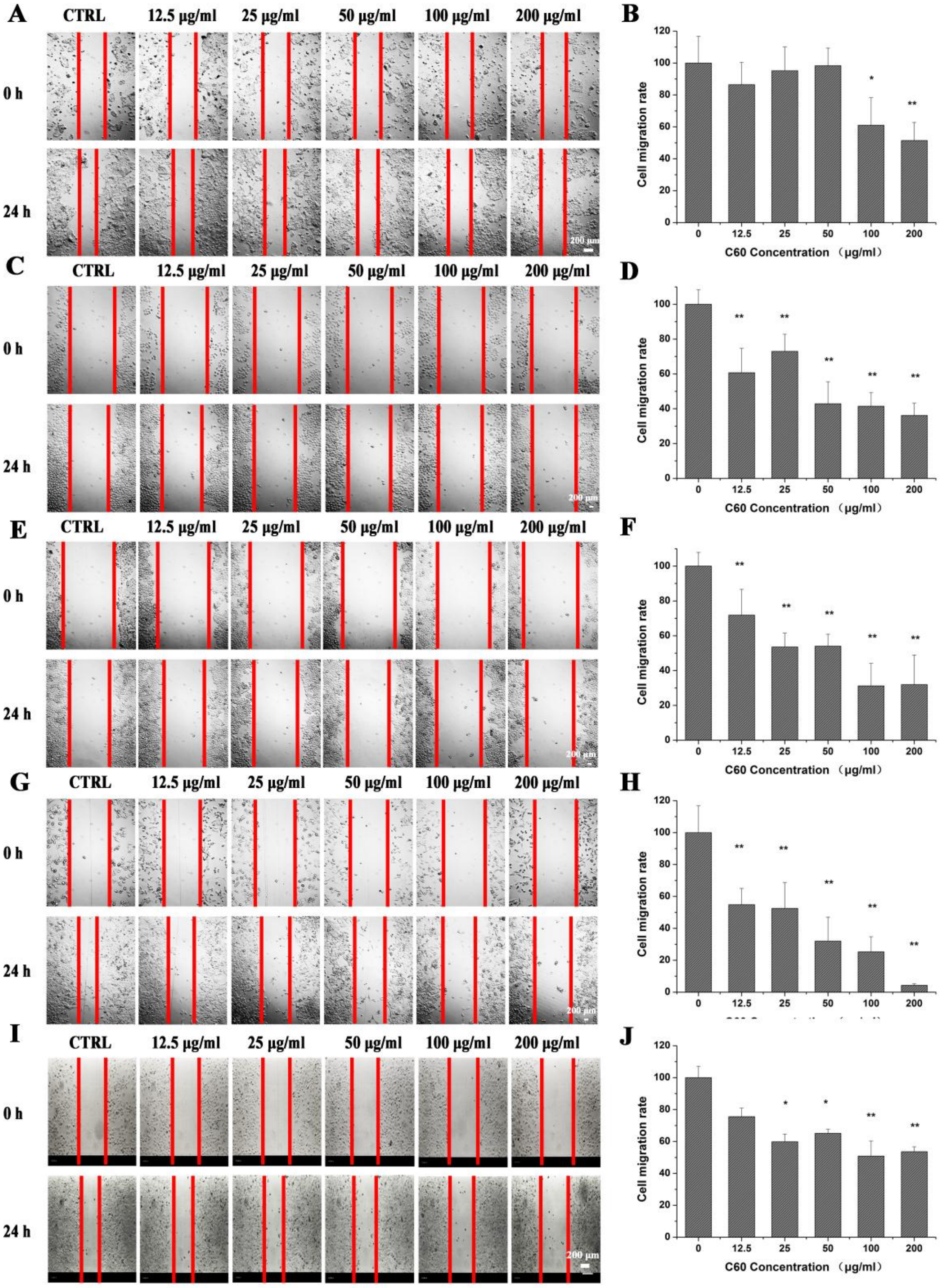

Supplement: Supplementary file 1 — Additional file 1. Additional figures. [file 12951_2018_380_MOESM1_ESM.pdf]
